# Utilizing the HiBiT System to Identify CARM1 Degraders for Targeted Cancer Therapy

**DOI:** 10.1021/acs.jmedchem.5c01863

**Published:** 2025-12-26

**Authors:** Megan Bacabac, Mingshan Hu, Fabao Liu, Eui-Jun Kim, Tanja Grkovic, Rohitesh Kumar, Rhone K. Akee, Isaac Hayes, Ramesh Mudududdla, Yidan Wang, Mason McGuire, Weiping Tang, Barry R. O’Keefe, Tim S. Bugni, Wei Xu

**Affiliations:** † McArdle Laboratory for Cancer Research, 5228 University of Wisconsin-Madison, Madison, Wisconsin 53705, United States; ‡ Natural Products Branch, Developmental Therapeutics Program, Division of Cancer Treatment and Diagnosis, 3421 National Cancer Institute, Frederick, Maryland 21702-1201, United States; § Molecular Targets Program, Center for Cancer Research, 3421 National Cancer Institute, Frederick, Maryland 21702-1201, United States; ∥ Natural Products Support Group, Leidos Biomedical Research, Inc., Frederick National Laboratory for Cancer Research, Frederick, Maryland 21702-1201, United States; ⊥ Lachman Institute for Pharmaceutical Development, School of Pharmacy, University of Wisconsin-Madison, Madison, Wisconsin 53705, United States; # Pharmaceutical Sciences Division, 5228 University of Wisconsin-Madison, Madison, Wisconsin 53705, United States

## Abstract

Preclinical studies validated coactivator-associated arginine methyltransferase 1 (CARM1) as a targetable therapeutic vulnerability, leading to the development of Proteolysis-Targeting Chimeras that specifically degrade CARM1. These compounds face significant translational challenges, including poor oral bioavailability and limited metabolic stability, which require extensive optimization. To identify more drug-like CARM1 degraders, we developed a high-throughput screening platform. We enabled antibody-free monitoring of CARM1 levels by fusing a HiBiT tag to CARM1 in MCF7 breast cancer cells. Complementation with LgBiT produces luciferase activity. Using this platform, we screened 1408 plant-derived natural product fractions to identify compounds that reduce CARM1 protein levels. This screen revealed two promising natural compounds, kusunokinin and exostemin, that specifically target CARM1 for degradation with selectivity over other protein arginine methyltransferases. Both compounds demonstrated functional anticancer activity, significantly inhibiting breast cancer cell colony formation and migration. Kusunokinin and exostemin represent lead compounds for developing next-generation CARM1-targeted therapeutics with enhanced translational potential.

## Graphical abstract

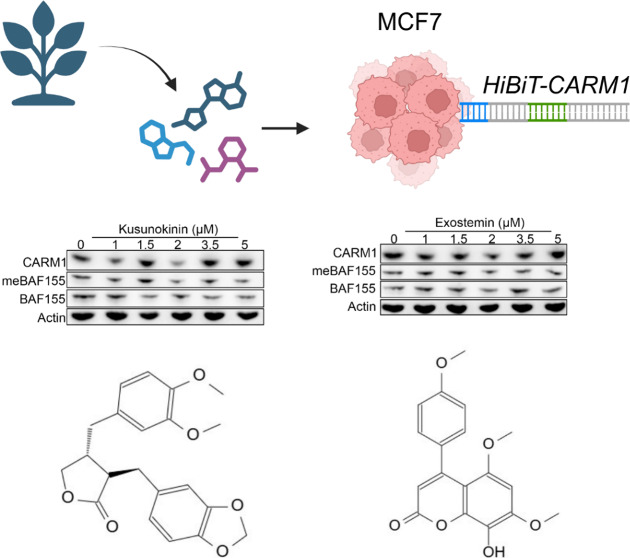

## Introduction

Coactivator-associated arginine methyltransferase 1 (CARM1) belongs to the protein arginine methyltransferase (PRMT) family and catalyzes asymmetrical dimethylation on arginine residues of histone and nonhistone proteins. This methylation activity drives tumor growth across multiple cancer types, including breast cancer, acute myeloid leukemia, gastric cancer, and small-cell lung cancer.^1^ In breast cancer, CARM1 mRNA shows consistent overexpression across all molecular subtypes and disease stages relative to normal tissue.^2,3^ The oncogenic role of CARM1 is mediated through methylation of various substrates that promote cancer progression. Notably, CARM1-mediated methylation of the BAF155 subunit within the SWI/SNF chromatin remodeling complex enhances metastasis while suppressing immune response in triple-negative breast cancer.^4^ Given the therapeutic potential of targeting CARM1, several small molecule inhibitors have been developed. Most of these inhibitors reduce the catalytic activity of CARM1 through a competitive mechanism. The inhibitors TP-064 and SKI-73 demonstrate efficacy in blocking cell invasion and migration, whereas EZM2302, compound 43, iCARM1, and YD1342 effectively reduce tumor growth and enhance immune cell infiltration.^5–10^ Despite these advances, small molecule inhibition of the methyltransferase activity of CARM1 has limitations in fully blocking its oncogenic functions.

Targeted protein degraders (TPDs) offer several advantages over traditional small molecule inhibitors. Unlike competitive inhibitors that must occupy an active site, TPDs can target previously “undruggable” proteins by binding to alternative sites, expanding the therapeutic landscape. Furthermore, TPDs address both the enzymatic and structural roles of target proteins. This distinction is particularly relevant for CARM1, which serves not only as a methyltransferase but also as a structural scaffold in DNA damage response and NF-κB dependent gene regulation.^11,12^ Consequently, degrading CARM1 would eliminate both its catalytic activity and any oncogenic scaffolding functions that cannot be addressed through active site inhibition alone, potentially enhancing their therapeutic efficacy.

The clinical translation of Proteolysis-Targeting Chimeras (PROTACs) has shown promising progress, exemplified by the estrogen receptor PROTAC vepdegestrant, developed by Arvinas and Pfizer, the first PROTAC to demonstrate clinical benefit in a Phase 3 trial (NCT05654623). However, PROTACs face significant pharmacokinetic challenges, including poor metabolic stability and limited oral bioavailability, both of which require extensive optimization.^13^ While we previously developed a potent CARM1-targeting PROTAC based on CARM1 inhibitor TP-064,^14^ we sought to identify CARM1 degraders with improved drug-like properties through unbiased high-throughput screening. Current CARM1 inhibitors and PROTACs utilize rational design using only the methyltransferase domain structure,^15^ whereas unbiased screening could reveal novel chemical scaffolds that engage unstructured domains of CARM1, potentially yielding compounds with superior pharmacological properties.

To enable high-throughput screening for compounds that induce CARM1 degradation, we developed a reporter assay system. Traditional antibody-based methods for quantifying endogenous CARM1 protein levels, such as Western blots, have inherent throughput limitations. We therefore employed CRISPR/Cas9 technology to engineer a cell line in which CARM1 is tagged with HiBiT, a small 11 amino acid peptide tag. Upon interaction with its complementation partner LgBiT, the resulting complex exhibits luciferase activity, enabling detection of CARM1 protein levels through luminescence-based luciferase assays.

Using this system, we screened 1408 natural product fractions from the National Cancer Institute’s Program for Natural Product Discovery (NPNPD) prefractionated library.^16^ We identified two natural products, kusunokinin and exostemin, that specifically decrease CARM1 protein levels over other PRMTs and effectively inhibit breast cancer cell migration and colony formation in vitro. These findings suggest that the assay system is suitable for the identification of natural product samples that can affect protein degradation end points and further that kusunokinin and/or exostemin may hold promise as lead compounds suitable for development as a targeted therapy for breast cancer. Essentially, our engineered cell line represents a valuable tool for discovering CARM1-targeting therapeutics that could enhance current breast cancer treatment strategies.

## Results

### Generation and Validation of MCF7-HiBiT-CARM1 Cell Line

To enable high-throughput screening for CARM1 degraders, we generated a HiBiT-CARM1 cell line using CRISPR/cas9-mediated knock-in technology. The HiBiT tag was inserted at the N-terminus of endogenous CARM1 in estrogen receptor-positive breast cancer cell line MCF7 (Figure 1A). Following nucleofection and cell expansion, single-cell cloning was performed to isolate individual clones. Three clones were selected and validated by functional luciferase assays. Upon addition of LgBiT and luciferase substrate, all three clones exhibited robust luminescent signals with ∼50–150-fold increase over background (Figure 1B). Clones 1 and 2 were validated by DNA sequencing.

**Figure 1:**
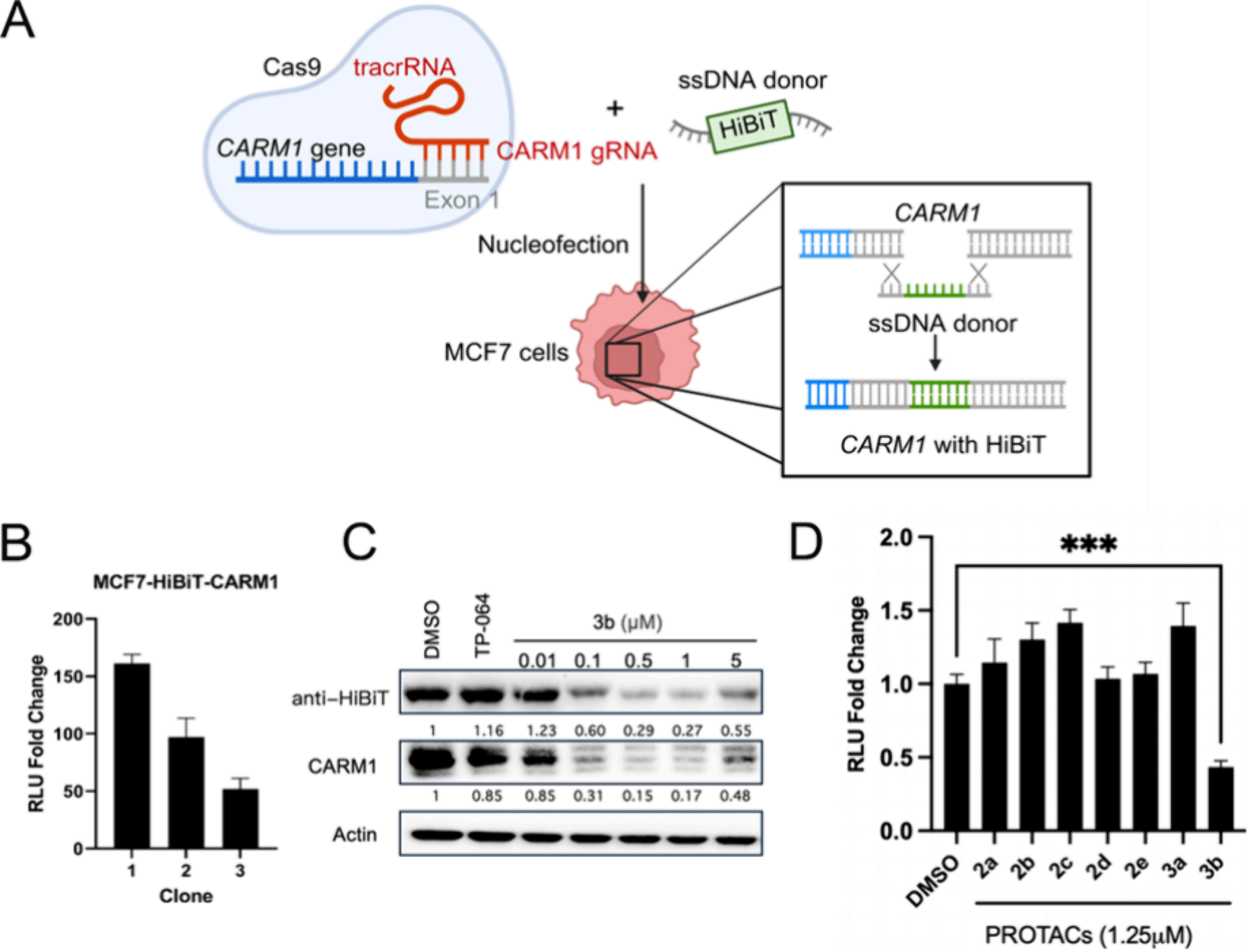
Generation and Validation of MCF7-HiBiT-CARM1 cell line. (A) Schematic of the strategy to knock-in HiBiT tag into *CARM1*. (B) Three clones of MCF7-HiBiT-CARM1 cells were grown to ∼90% confluency. The relative luminescence compared to wild-type MCF7 cells were assessed by a luciferase assay. (C) MCF7-HiBiT-CARM1 cells were treated with DMSO vehicle, 10 μM CARM1 inhibitor TP-064, or the indicated concentrations of PROTAC 3b for 24 h. Protein expression levels of HiBiT-tagged CARM1 were assessed by immunoblotting. (D) MCF7-HiBiT-CARM1 cells were treated with DMSO vehicle or 1.25 μM of various CARM1 PROTACs for 48 h. Relative HiBiT-CARM1 levels were assessed by a luciferase assay. Mean ± SD, *n* = 3, ***P < 0.001. *P* values were calculated using two-tailed unpaired Student’s *t* tests.

We further validated successful HiBiT integration through immunoblotting analysis. Treatment with the established CARM1 PROTAC 3b^14^ resulted in comparable reduction of both anti-HiBiT and anti-CARM1 signals, confirming effective degradation of the tagged protein (Figure 1C). To assess the system’s suitability for screening applications, we conducted a pilot screen using a panel of previously characterized PROTACs with known CARM1 degradation activity.^14^ As expected, compound 3b produced the greatest reduction in HiBiT signal among the tested compounds (Figure 1D). These results demonstrate successful generation of a functional HiBiT-CARM1 reporter system capable of detecting endogenous CARM1 protein levels for high-throughput degrader screening.

### Optimization of High-Throughput Screen with MCF7-HiBiT-CARM1 Cells

To enable large-scale screening, we systematically optimized assay conditions for the HiBiT-CARM1 cell line in a 384-well plate format. Initial cell density optimization involved seeding cells at four different concentrations and assessing their viability after 72 h using CellTiterGlo. While cell viability remained robust across most densities, a slight decrease was observed at the highest concentration (10,000 cells/well), leading us to focus on the 2000–5000 cells/well range (Figure 2A).

**Figure 2:**
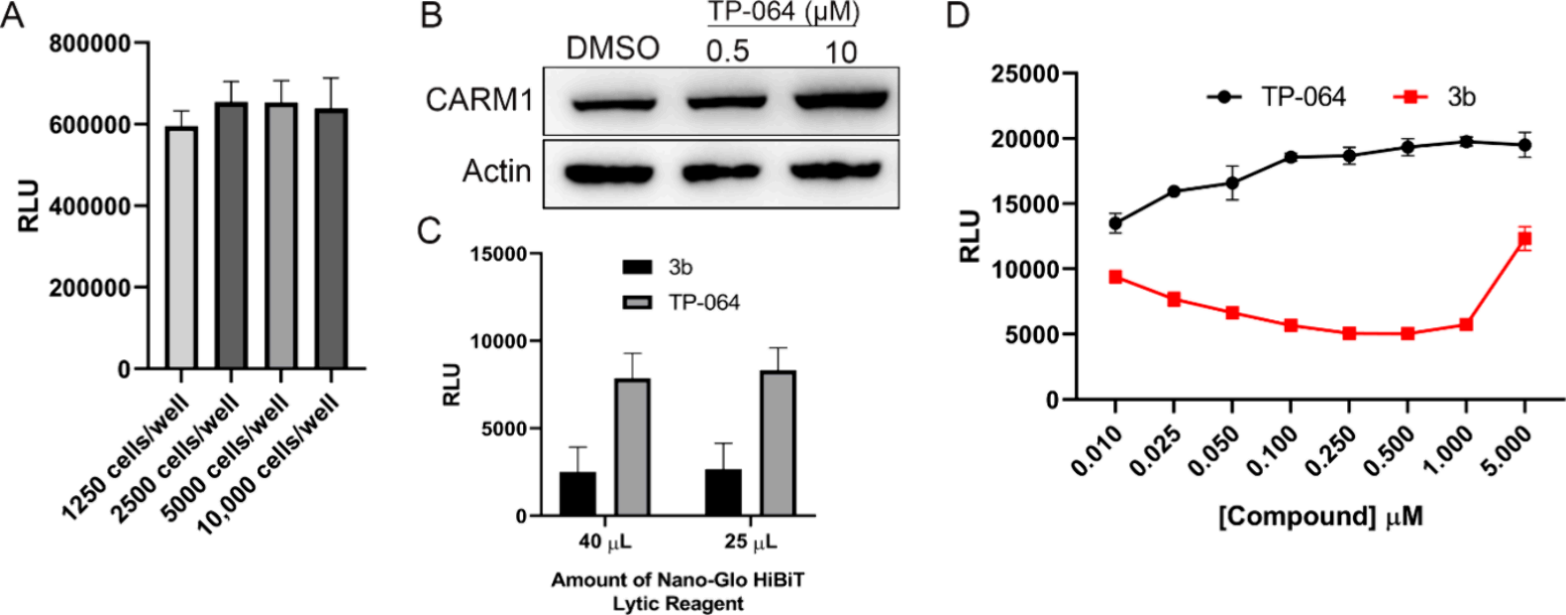
Optimization of the MCF7-HiBiT-CARM1 line for high-throughput screening. (A) MCF7-HiBiT-CARM1 cells were seeded at different densities into a 384-well plate. After 72 h, cell viability was assessed with CellTiterGlo. (B) Wild-type MCF7 cells were treated with DMSO or the indicated doses of TP-064 for 24 h. CARM1 levels were assessed by immunoblotting. (C) MCF7-HiBiT-CARM1 cells were treated with 1 μM 3b and 10 μM TP-064 for 48 h. At end point, HiBiT-CARM1 levels were assessed by luciferase assay. (D) MCF7-HiBiT-CARM1 cells were treated with indicated doses of TP-064 or 3b for 48 h. Relative HiBiT-CARM1 levels were assessed by a luciferase assay.

For assay controls, we selected the established CARM1 PROTAC compound 3b as a positive control for protein degradation. Interestingly, treatment with the CARM1 inhibitor TP-064 resulted in increased CARM1 protein levels as we recently reported,^3^ making it suitable as a negative control (Figure 2B). We evaluated assay performance using *Z*′ factor analysis across three seeding densities (2000, 3000, and 4000 cells/well) with DMSO as a negative control and TP-064 as a positive control for CARM1 levels. Optimal assay performance was achieved at 2000 cells/well using TP-064, yielding the highest *Z*′ factor (Table 1).

**Table 1:** *Z*′ Factors Scores in 384-Well Plate Format

cells/well	2000	3000	4000
DMSO	0.613	0.498	0.621
TP-064	0.919	0.806	0.766

Reagent optimization revealed that the HiBiT detection system was highly sensitive, as reducing the volumes of lytic buffer, luciferase substrate, and LgBiT peptide to half the manufacturer’s recommended amount maintained equivalent luminescent signal intensity (Figure 2C). Dose–response analysis of control compounds confirmed that 1 μM concentrations were optimal with compound 3b showing the expected hook effect at higher concentrations, while TP-064 demonstrated dose-dependent increases in CARM1 levels (Figure 2D).

The exceptional assay sensitivity enabled further miniaturization to the 1536-well format. Using 1000 cells/well with DMSO as a negative control and TP-064 as a positive control for CARM1 levels, we achieved a *Z*′ factor score of 0.415 and 0.555, respectively (Table 2), confirming the robustness of this system for high-throughput CARM1 degrader screening.

**Table 2:** *Z*′ Factor Scores in 1536-Well Plate Format

cells/well	500	1000
DMSO	0.334	0.415
TP-064	0.512	0.555

### Screening the NPNPD Prefractionated Library for CARM1 Degraders

We selected the NPNPD library for CARM1 degrader screening for several reasons. Natural products represent a historically rich yet underexplored source of therapeutic compounds, with many current anticancer agents derived from natural origins, including widely used chemotherapy drugs paclitaxel and doxorubicin. A comprehensive 2020 analysis demonstrated that between January 1981 and September 2019, 41% of globally approved small molecule drugs are naturally inspired.^17^ Furthermore, previous studies have identified natural products capable of inhibiting CARM1 activity, suggesting the potential for discovering natural product-derived CARM1 degraders.^18,19^

For the primary screen, HiBiT-CARM1 cells were seeded in 1536-well plates and allowed to adhere overnight before treatment with single doses of control compounds (TP-064 and 3b), and 1408 natural product fractions. Following 48-h incubation, cells were assayed for luciferase activity in parallel with a CellTiterGlo viability counter screen to exclude fractions causing HiBiT signal depletion through cytotoxicity (Figure 3A).

**Figure 3:**
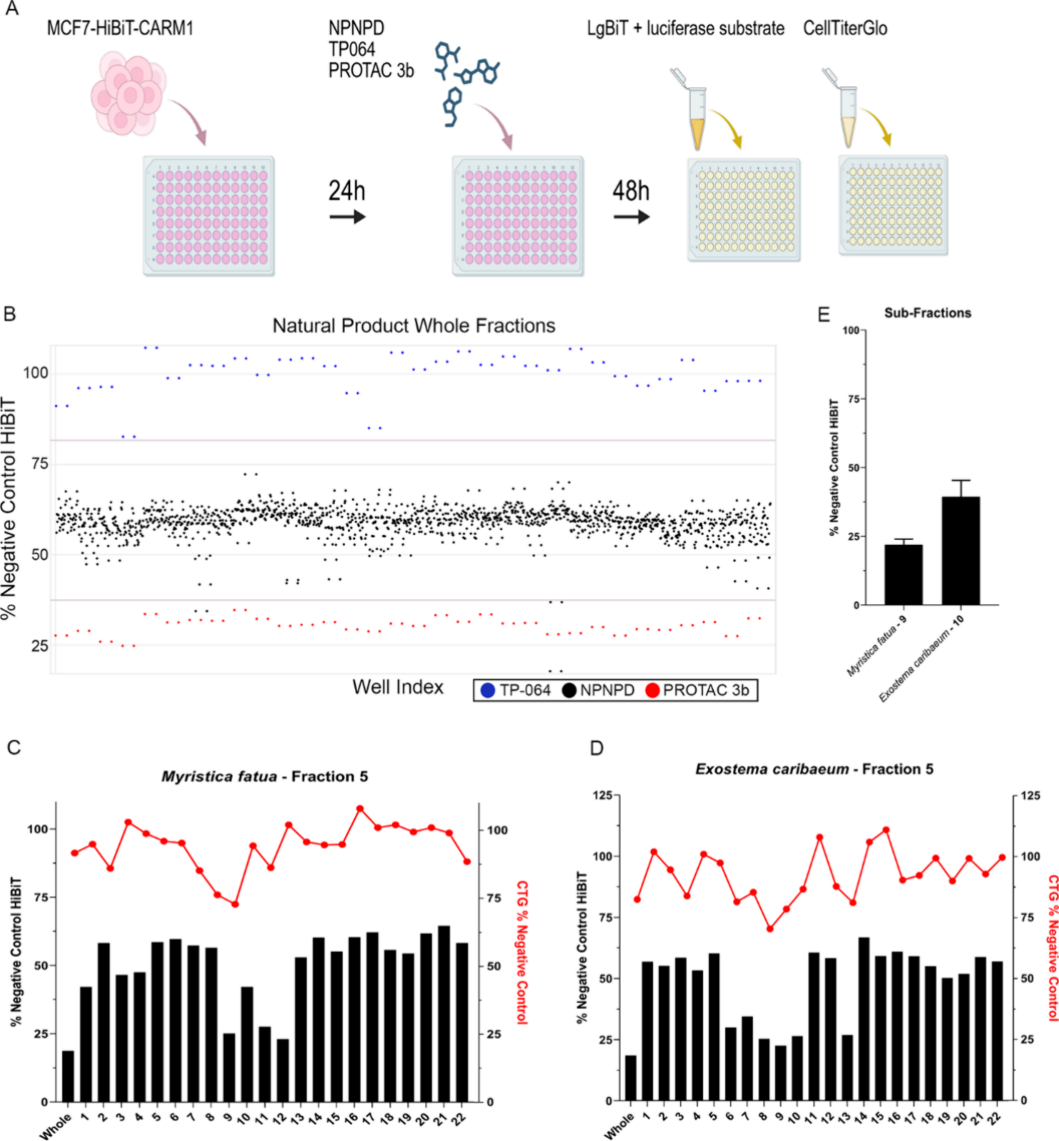
High-throughput screening of a prefractionated natural products library for CARM1 degraders. (A) Schematic of high-throughput screen workflow. (B) MCF7-HiBiT-CARM1 cells were treated with 5 ng/μL of 1408 mixed natural product fractions (black dots). TP-064 and 3b treated cells are represented by blue and red dots, respectively. Purple lines indicate ± 2 standard deviations. (C,D) MCF7-HiBiT-CARM1 cells were treated with 10 ng/μL of the whole natural product fraction or a subfraction (1–22) for 48 h. HiBiT-CARM1 levels were detected by luciferase assay (black bars) and cell viability was detected using Cell Titer Glo (red dots). Activity of two whole fractions and all their subfractions are represented. (E) MCF7-HiBiT-CARM1 cells were treated with 10 ng/μL of two subfractions that were sent out for purification. HiBiT-CARM1 levels were detected by luciferase assay.

The screen demonstrated robust performance with a *Z*′ factor score of 0.66 and a signal-to-noise ratio of 3.3, where the luminescent signal was represented as a scatterplot (Figure 3B). Hit selection criteria required fractions to maintain greater than 60% cell viability while reducing HiBiT signal below 40%, yielding a 0.4% positive-hit rate. The top 11 hits were advanced to secondary screening.

Secondary purification using automated systems developed in the NPNPD generated 22 subfractions from each of the 11 primary hits,^20^ which were then evaluated using identical HiBiT and viability assays (Figure 3C,D). The 11 most promising subfractions were validated by a subsequent replicate, then underwent tertiary purification and structural characterization (Figure 3E).

### Characterization of Two Putative Natural CARM1 Degraders

Structural elucidation was completed for 11 compounds, followed by dose-dependent efficacy assessment using luciferase assays. This analysis identified two lead candidates: *Myristica fatua*—9 and *Exostema caribaeum*—10 (Figure 4A,B). Cell viability after treatment with these two fractions was also assessed and consistent with the findings in Figure 3, neither of these fractions significantly decreased cell viability (S1A,B).

**Figure 4:**
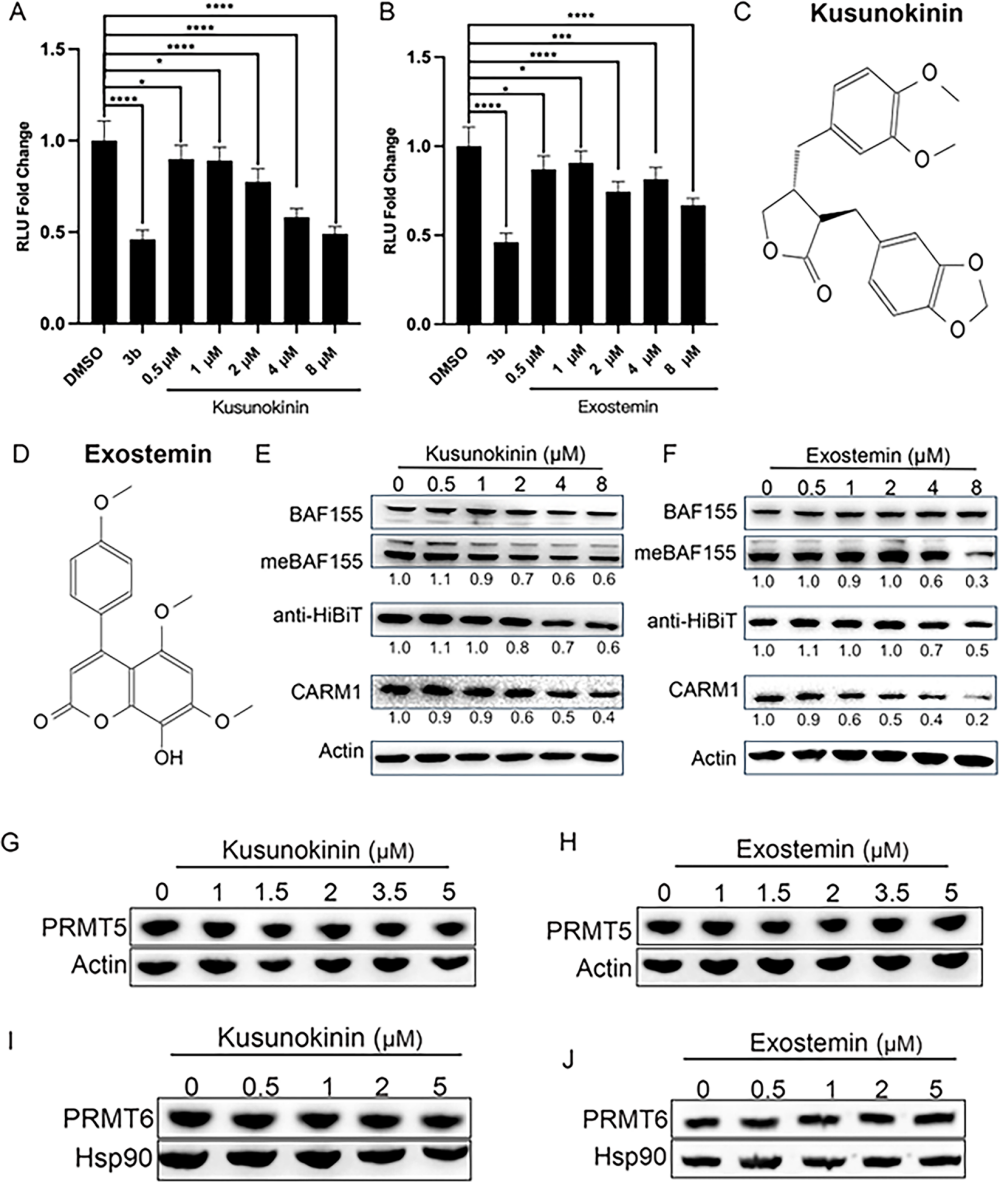
Kusunokinin and exostemin are selective CARM1 degraders. (A,B) MCF7-HiBiT-CARM1 cells were treated with 1.25 μM 3b or the indicated doses of *Myristica fatua*—9 (A) or *Exostema caribaeum*—10 (B) for 72h. HiBiT-CARM1 levels were assessed by a luciferase assay. Mean ± SD, *n* = 5, *P < 0.05, **P < 0.01, ***P < 0.001, and ****P < 0.0001. *P* values were calculated using two-tailed unpaired Student’s *t* tests. (C) Chemical structure of kusunokinin. (D) Chemical structure of exostemin. (E,F) MCF7-HiBiT-CARM1 cells were treated with the indicated doses of kusunokinin (E) or exostemin (F) for 72 h. CARM1, methyl-BAF155, and total BAF155 levels were detected by immunoblotting. (G,H) MCF7 cells were treated with the indicated doses of kusunokinin (G) or exostemin (H) for 72 h. PRMT5 levels were assessed by immunoblotting. (I,J) MCF7 cells were treated with the indicated doses of kusunokinin (I) or exostemin (J) for 24 or 48 h, respectively. PRMT6 levels were assessed by Western blot.

The first compound was identified to be (−)-kusunokinin (Figure 4C), a lignan originally extracted from the *Myristica fatua* of the *Myristicaceae* family and originally discovered in 1977.^21^ The second compound was determined to be exostemin (Figure 4D), a neoflavonoid isolated from *Exostema caribeaum* of the *Rubiaceae* family, initially discovered in 1969.^22^

We validated CARM1 protein reduction by both compounds in MCF7-HiBiT-CARM1 cells using Western blot analysis. Consistent with CARM1 degradation, levels of methyl-BAF155, a CARM1-specific substrate, decreased proportionally with CARM1 protein levels (Figure 4E,F). However, both compounds required relatively high concentrations (∼2–5 μM) to achieve these effects, indicating modest potency compared to rationally designed PROTAC degraders.

To assess selectivity within the protein arginine methyltransferase family, we examined effects on PRMT5 and PRMT6 in wild-type MCF7 cells. Neither kusunokinin nor exostemin affected these related enzymes at the concentrations tested (Figure 4G–J). However, comprehensive proteome-wide selectivity profiling was not performed.

### Mechanism of CARM1 Protein Depletion

To elucidate the mechanism underlying CARM1 protein reduction, we first examined the transcriptional effects. Treatment with multiple doses of either kusunokinin or exostemin failed to significantly alter CARM1 mRNA levels (Figure 5A,B), indicating post-transcriptional regulation. We next investigated protein stability using cycloheximide chase assays with or without the proteasome inhibitor MG-132. MCF7 cells were treated with vehicle (DMSO), PROTAC 3e (a metabolically stable analog of 3b with similar activity),^14^ kusunokinin, or exostemin. The control DMSO experiment demonstrated endogenous CARM1 half-life when new protein synthesis is blocked. An experiment with 3e demonstrated the expected CARM1 degradation over time, which was prevented by MG-132 cotreatment^14^ (Figure 5C,D). Exostemin treatment resulted in CARM1 reduction following cycloheximide exposure, while MG132 cotreatment completely prevented this degradation (Figure 5E). These results indicate that exostemin promotes CARM1 degradation through the ubiquitin-proteasome system (UPS). In contrast, kusunokinin-treated cells showed CARM1 reduction at 24 h with cycloheximide that persisted even with MG132 cotreatment (Figure 5F). This MG-132-insensitive degradation suggests that kusunokinin depletes CARM1 through a nonproteasome pathway, potentially involving lysosome or other degradation mechanisms.

**Figure 5:**
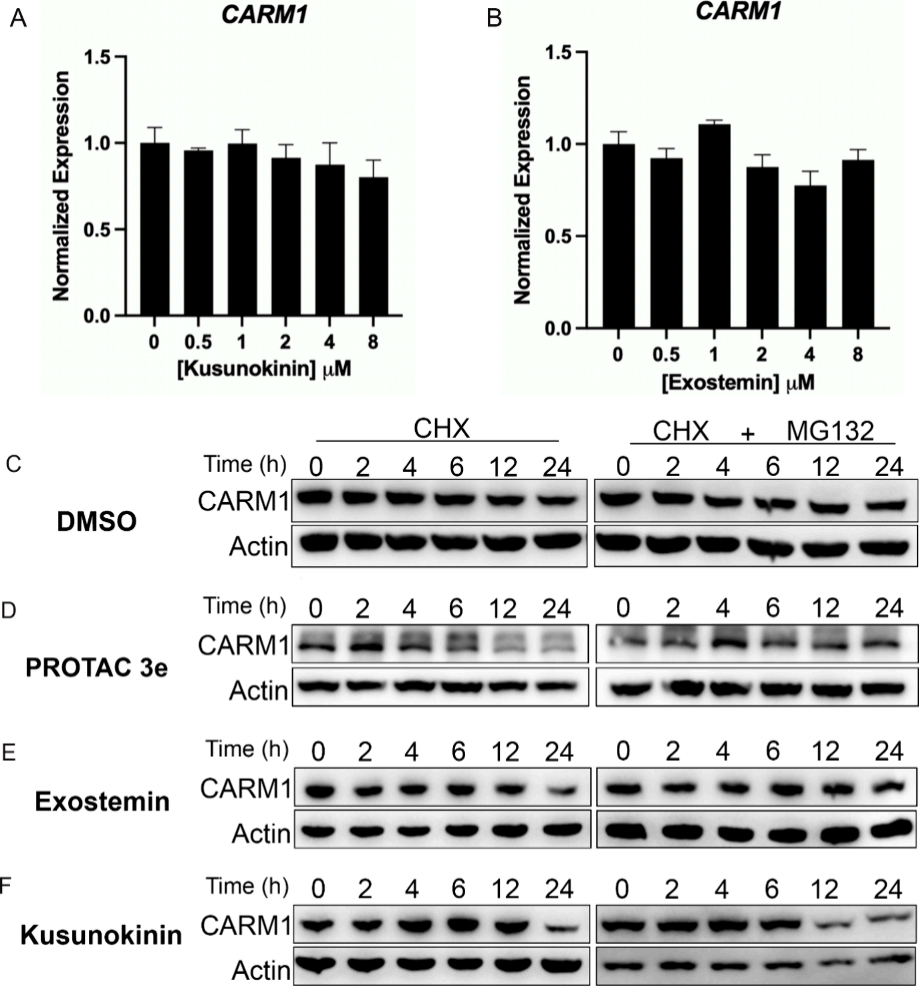
Treatment with kusunokinin and exostemin leads to CARM1 protein destabilization without affecting CARM1 mRNA levels. (A,B) Parental MCF7 cells were treated with the indicated doses of kusunokinin (A) or exostemin (B) for 72 h. RNA was extracted to cells and expression of *CARM1* mRNA was assessed by qRT-PCR. (C–F) MCF7 cells were treated with DMSO (C), 0.5 μM 3e (D), 3.5 μM exostemin (E), or 3.5 μM kusunokinin (F), then cotreated with cycloheximide only or cycloheximide and 7.5 μM MG132 for the indicated time points. CARM1 levels were assessed by immunoblotting.

To test whether kusunokinin acts through the lysosomal pathway, MCF7-HiBiT-CARM1 cells were cotreated with kusunokinin and bafilomycin A1, a lysosomal inhibitor that blocks autophagic degradation by preventing lysosome acidification. The dose of bafilomycin used was determined from the IC50 value (Figure S2). Kusunokinin treatment alone resulted in decreased CARM1 and HiBiT levels; however, bafilomycin cotreatment rescued CARM1 protein levels (Figure 6A). These findings demonstrate that kusunokinin-mediated CARM1 degradation operates through the lysosomal pathway rather than the UPS, distinguishing its mechanism from that of exostemin and conventional PROTACs.

**Figure 6:**
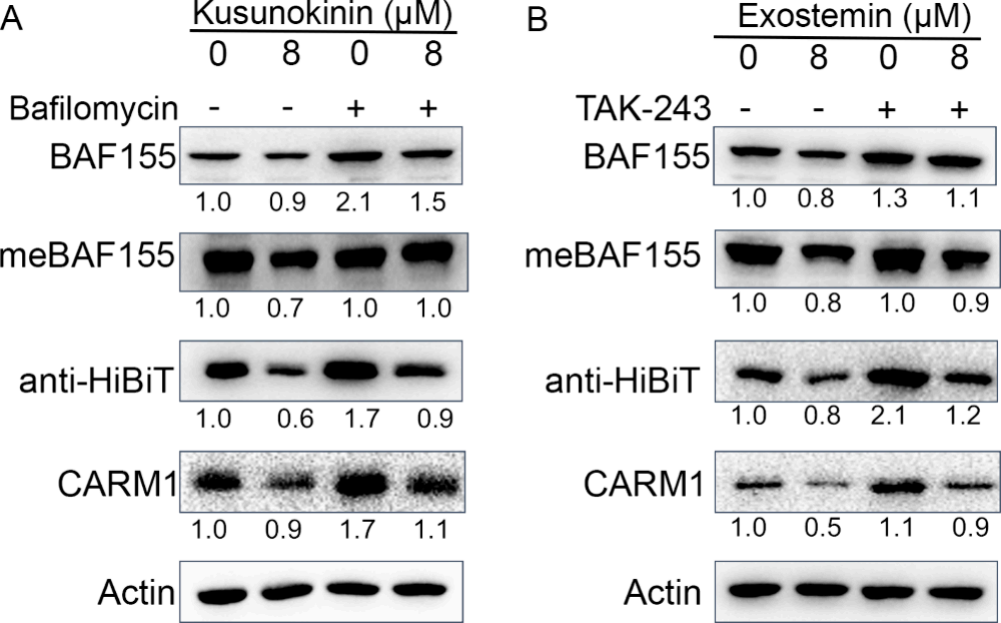
Kusunokinin and exostemin degrade CARM1 through different degradation pathways. (A) MCF7-HiBiT-CARM1 cells were treated for 72 h with 8 μM kusunokinin with or without 0.02 μM bafilomycin. BAF155, meBAF155, HiBiT-CARM1, and CARM1 levels were assessed by immunoblotting. (B) MCF7-HiBiT-CARM1 cells were treated for 72 h with 8 μM exostemin with or without 0.03 μM TAK-243. BAF155, meBAF155, HiBiT-CARM1, and CARM1 levels were assessed by immunoblotting.

To further confirm that exostemin-mediated CARM1 degradation requires ubiquitination, we cotreated MCF7-HiBiT-CARM1 cells with exostemin and TAK-243, a ubiquitin-activating enzyme (E1) inhibitor that blocks the ubiquitin cascade upstream of E3 ligase engagement. The dose of TAK-243 used was determined from the IC50 of 0.6 μM (Figure S3). Consistent with the MG-132 coexperiment results (Figure 5E), TAK-243 cotreatment rescued CARM1 and HiBiT levels that were otherwise depleted by exostemin alone (Figure 6B). These results confirm that exostemin promotes CARM1 degradation through a ubiquitin-dependent, proteasome-mediated mechanism.

To further validate the pathway specificity of each compound, we performed reciprocal inhibitor experiments. Co-treatment of exostemin with bafilomycin failed to rescue CARM1 levels (Figure S4A), confirming that exostemin does not operate through the lysosomal pathway. Conversely, TAK-243 cotreatment with kusunokinin did not rescue CARM1 degradation (Figure S4B), demonstrating that kusunokinin acts independently of the ubiquitin-proteasome system. Together, these cross-inhibitor experiments establish distinct degradation mechanisms: exostemin functions through UPS-mediated proteolysis, while kusunokinin promotes lysosome-dependent CARM1 degradation.

### Treatment with Kusunokinin and Exostemin Inhibits Colony Formation and Cell Migration

Since previous studies demonstrated that temporary CARM1 degradation does not inhibit cell proliferation,^4,14^ while it does inhibit colony formation, a measure of anchorage-independent growth and tumorigenic potential, we investigated whether kusunokinin and exostemin inhibit colony formation. MCF7 cells were treated with increasing concentrations of PROTAC 3e, kusunokinin, or exostemin in colony formation assays. PROTAC 3e achieved 50% inhibition of colony formation at 0.5 μM, while kusunokinin and exostemin required 2 and 5 μM, respectively, to achieve comparable inhibition levels (Figure 7A,B). These results demonstrate that CARM1 degradation, including that elicited by the newly identified compounds, effectively suppresses anchorage-independent growth.

**Figure 7:**
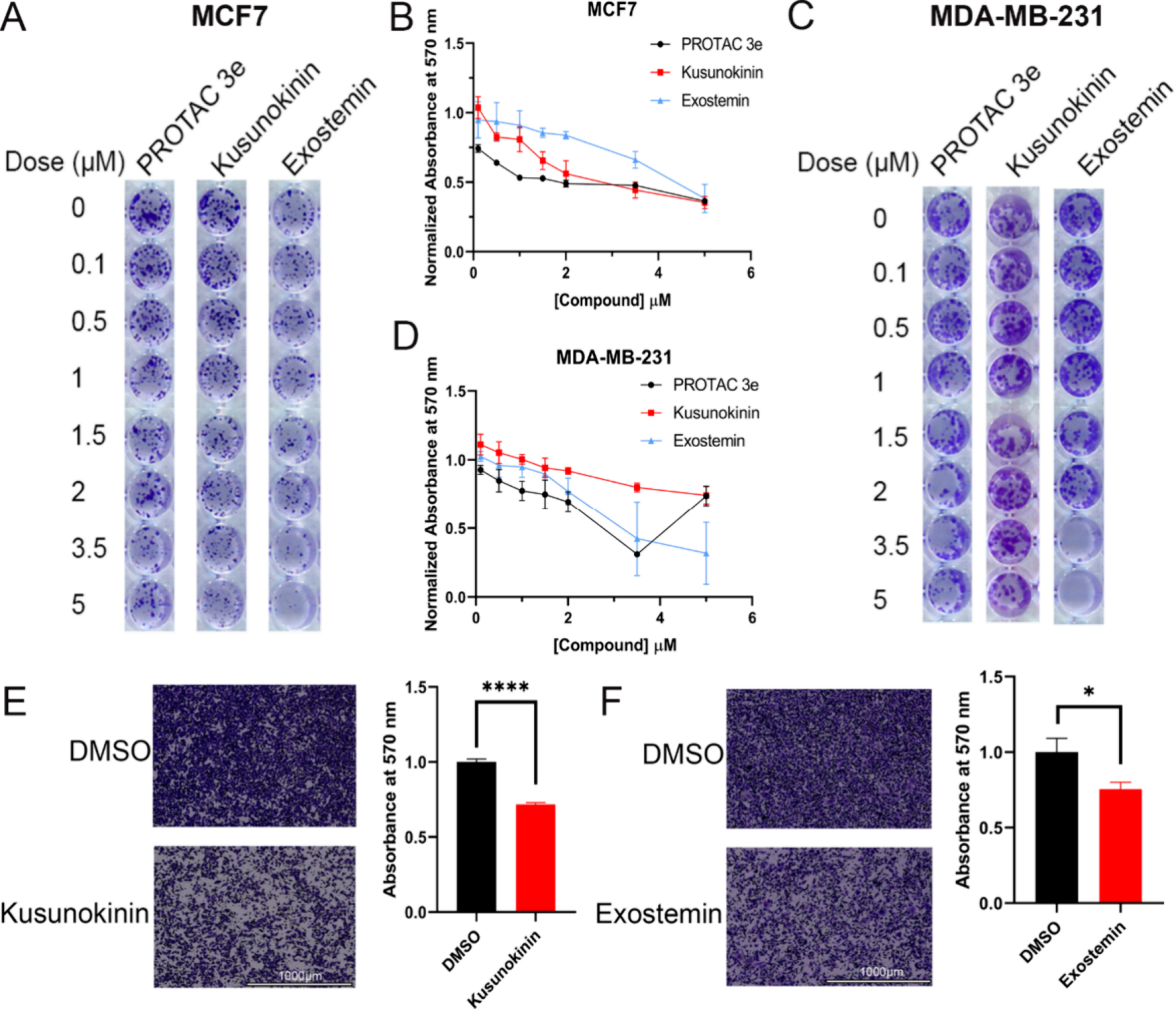
Biological effects of kusunokinin and exostemin. (A) MCF7 cells were treated with the indicated doses of 3e, kusunokinin, and exostemin for 2 weeks. Formed colonies were fixed and stained with 0.1% crystal violet. (B) Colonies were quantified by resolving the crystal violet stain with 10% acetic acid. (C) MDA-MB-231 cells were treated with the indicated doses of 3e, kusunokinin, and exostemin for 2 weeks. Formed colonies were fixed and stained with 0.1% crystal violet. (D) Colonies were quantified by resolving the crystal violet stain with 10% acetic acid. (E,F) MDA-MB-231 cells were treated with 2 μM kusunokinin (E) or 3.5 μM exostemin for 24 h, then seeded into Transwells. Cells were allowed to migrate for 16 h. Migrated cells were fixed and stained with 1% crystal violet. Migrated cells were quantified by resolving the crystal violet stain in 10% acetic acid (right panels).

Given our previous findings that CARM1 degradation also inhibits cell migration,^14^ we next evaluated the migratory effects of these natural product degraders. Since MCF7 cells exhibit limited migratory capacity, we utilized the highly motile triple-negative breast cancer cell line, MDA-MB-231 for these studies. Both kusunokinin and exostemin maintained their ability to inhibit colony formation in MDA-MB-231 cells (Figure 7C,D), although higher concentrations were required as compared to 3e, as observed in MCF7 cells. Subsequent migration assays revealed that treatment with either kusunokinin or exostemin significantly reduced MDA-MB-231 cell motility (Figure 7E,F). These functional outcomes demonstrate that natural product-mediated CARM1 degradation effectively inhibits key oncogenic processes associated with CARM1 activity, including anchorage-independent growth and cellular migration, implicating therapeutic potential for these compound scaffolds in breast cancer treatment.

## Discussion

This study establishes a robust screening platform for identifying CARM1 degraders and demonstrates their therapeutic efficacy in breast cancer cells. We utilized the well-established HiBiT technology due to its ability to rapidly assess degradation of target proteins.^23,24^ Our approach leverages two underexplored areas of drug discovery: natural products as a source of bioactive compounds and targeted protein degradation as an emerging therapeutic modality. While natural products have been incorporated into the targeted protein degradation field primarily as warheads or E3 ligase ligands in PROTAC design, our work represents a significant advancement by identifying molecular glue-like degraders that promote specific protein degradation without requiring synthetic chemical linkers.

Many previous compounds put forward as CARM1 inhibitors were primarily S-adenosyl methionine mimetics that competitively inhibited CARM1 methyltransferase activity.^25^ Our screen identified two structurally and functionally distinct compounds: exostemin, a neoflavonoid with limited prior characterization, and kusunokinin, a well-studied lignan with documented anticancer activities. Kusunokinin has demonstrated potent antiproliferative effects in breast cancer models, with synthetic kusunokinin inhibiting MCF7 and MDA-MB-231 cell viability at IC_5__0_ values of 4.3 and 7.6 μM, respectively.^26^ The compound induces G2/M cell cycle arrest and apoptosis through multiple mechanisms, including topoisomerase II inhibition, Bcl-2 downregulation, and upregulation of p53, p21, and cytochrome C.^27^ Our findings that kusunokinin inhibits colony formation and cell migration in breast cancer cells align with these established antiproliferative effects.^26,27^

The anticancer mechanism of kusunokinin has been reported to involve partial binding to Colony Stimulating Factor 1 Receptor (CSF1R), resulting in AKT pathway suppression and downstream effects including Cyclin D1 reduction in MCF7 cells.^26^ Additionally, computational modeling predicts kusunokinin binding to aldo-keto reductase family 1 member B1 (AKR1B1),^28^ though experimental validation of this interaction remains to be determined. Natural products commonly exhibit polypharmacology. Thus, kusunokinin and exostemin likely engage multiple cellular targets, representing proof-of-concept natural product CARM1 degraders with modest potency rather than optimized selective tools.

Our mechanistic investigations reveal fundamentally different degradation pathways for the two lead compounds, highlighting the diversity of natural product-mediated protein degradation mechanisms. Exostemin appears to function through the ubiquitin-proteasome system, as evidenced by MG-132-mediated rescue of CARM1 protein levels (Figure 5E) and TAK-243-mediated rescue of CARM1 and HiBiT-CARM1 levels when cotreated with exostemin (Figure 6B). This suggests that exostemin may act as a molecular glue, potentially bridging CARM1 with an E3 ubiquitin ligase to facilitate targeted degradation. The identity of the specific E3 ligase remains to be determined, presenting an opportunity to expand the current toolkit of E3 ligases utilized in targeted protein degradation. Understanding whether exostemin engages established E3 ligase networks or facilitates novel ligase partnerships could significantly advance the field by providing new scaffolds for degrader development.

In contrast, kusunokinin-mediated CARM1 degradation occurs through a proteasome-independent mechanism, as degradation persists despite MG132 treatment (Figure 5F) but can be rescued by bafilomycin A1 cotreatment (Figure 6A). This observation strongly suggests involvement of alternative proteolytic pathways, most likely autophagy-lysosome-mediated degradation. This finding is particularly intriguing given that kusunokinin also decreases Topoisomerase II and STAT3 protein levels in MCF7 cells,^26^ suggesting it may function as a broad-spectrum protein degrader or indirectly regulate protein stability through upstream signaling pathways.

The lysosomal pathway has gained attention in targeted protein degradation through platforms like lysosome-targeting chimeras (LYTACs), which have shown particular promise for degrading extracellular and membrane proteins. However, lysosomes also process intracellular proteins through multiple routes, including chaperone-mediated autophagy and macroautophagy. Elucidating the precise mechanism by which kusunokinin promotes CARM1 degradation will require a comprehensive investigation of these various lysosomal pathways.

Our findings underscore the continued importance of discovery of natural products as tool and potential lead compounds in pharmaceutical development, particularly in the context of precision oncology. For triple-negative breast cancer, CARM1 represents a compelling therapeutic target given its overexpression and its role in promoting metastasis and immune evasion. While synthetic CARM1 PROTACs have demonstrated potent degradation activity, the discovery of kusunokinin and exostemin as natural CARM1 degraders offers unique advantages. As these are naturally derived products, the potential for *in vivo* toxicity decreases. These compounds may serve as valuable pharmacophores for structure-based drug design and medicinal chemistry optimization. The natural product scaffolds provide evolutionarily refined starting points that can be systematically modified to enhance potency, selectivity, and drug-like properties while maintaining their inherent biocompatibility. A recent review has indicated that the clinical progression of natural product chemotypes is more successful than that achieved by purely synthetic molecules.^29,30^ This approach may ultimately yield more effective, naturally inspired therapeutic agents specifically tailored for CARM1-driven malignancies, representing a promising convergence of traditional natural product medicine with modern precision oncology strategies.

## Experimental Section

### Cell Culture

MCF7 cells (clone WS8) were provided by Dr. V. Craig Jordan and MDA-MB-231 cells were purchased from the American Type Culture Collection (ATCC). MCF7 cells were cultured in Dulbecco’s modified Eagle’s Medium (DMEM) supplemented with 10% FBS (Cytiva) and 1% penicillin-streptomycin. MDA-MB-231 cells were cultured in RPMI 1640 supplemented with 10% FBS and 1% penicillin-streptomycin. All cells were cultured at 37 °C in a humidified incubator at 5% CO_2_.

### Generation of MCF7-HiBiT-CARM1 Cell Line

To generate this cell line, a guide RNA (gRNA) targeting the N-terminus of CARM1 and a single-strand DNA (ssDNA) oligonucleotide containing microhomology arms to *CARM1* and the sequence for HiBiT were designed. The gRNA was incubated with purified Cas9 protein to form a ribonucleoprotein (RNP) complex. MCF7 cells were prepared for nucleofection using the SE Line X Kit S (Lonza). The RNP complex and ssDNA oligo were added to MCF7 cells, then loaded into the provided nucleofector 16-well cassette. Cells were electroporated in the 4D-Nucleofector X Unit using the manufacturer’s protocol for MCF7 cells. Cells were expanded in culture, then single-cell seeded into 96-well plates to isolate single clones.

gRNA: AGCCGGATCTAAGATGGCAG

ssDNA oligo: GGCGGCCTGG​GCCCGGGCGC​AGCGGCGGCGGC​GGCGGGGCCT​GGAGCCGGATCT​AAGATGGTGAG​CGGCTGGCGG​CTGTTCAAGAA​GATTAGCGCA​GCGGCGGCG​GCGGCGGTGGG​GCCGGGCGCGG​GCGGCGCGGGG​TCGGCGGTCC​CGGGC

### Nano-Glo HiBiT Lytic Assay

Cells were seeded using the Microflo Select Reagent Dispenser (BioTek) into white-walled multiwell plates. At end point, LgBiT protein (1:100) and Nano-Glo HiBiT lytic substrate (1:50) diluted in Nano-Glo HiBiT lytic reagent (Promega) were added to wells using the Microflo Select. Plates were incubated in the dark at room temperature for 10 min on a rotator. Luminescence was read on the Victor x5 2030 Multilabel Reader (PerkinElmer) or the PHERAstar FS (BMG Labteach).

### Western Blotting

Proteins were resolved in 8% SDS–PAGE gels and transferred to nitrocellulose membranes using the Bio-Rad Turbo Blot (Bio-Rad). Blots were blocked in 5% skim milk in PBST (PBS and 0.1% Tween 20) for 1 h, then incubated with primary antibodies in 2.5% skim milk in PBST at 4 °C overnight. Primary antibodies used in this study are anti-CARM1, antime-BAF155,^31^ anti-BAF155 (D7F8S, Cell Signaling Technology), anti-PRMT5 (D5P2T, Cell Signaling Technology), anti-PRMT6 (D-5, Santa Cruz Biotechnology), anti-HiBiT (Promega), anti-β-Actin (ABclonal Technology, Sigma-Aldrich), and anti-Hsp90 (H-114, Santa Cruz Biotechnology). Blots were washed in PBST for 30 min, then incubated with horse radish peroxidase-labeled secondary antibodies diluted in 2.5% skim milk for 1–2 h at room temperature. Secondary antibodies were washed off in PBST for 30 min on a shaker at room temperature, then incubated with SuperSignal West Pico ECL (Thermo Fisher Scientific). Blots were exposed using an Azure 600 imaging system (Azure Biosystems).

### Cell Viability Assay

Cells were seeded using the Microflo Select Reagent Dispenser (BioTek) into white-walled multiwell plates. At end point, CellTiter-Glo substrate in the provided buffer (Promega) was added to wells using the Microflo Select. Plates were incubated in the dark at room temperature for 10 min on a rotator. Luminescence was read on the PHERAstar FS (BMG Labtech).

### Cell Counting

MCF7-HiBiT-CARM1 cells were seeded 1000 cells/well in 100 μL of DMEM into the wells of a 96-well plate. Cells were treated with DMSO, kusunokinin, or exostemin. The plate was imaged after 48 h of treatment using the High-Contrast Brightfield Kit for Label-Free Cell Counting (BioTek) on the Lionheart FX Automated Microscope (Bio-Tek).

### RT-qPCR

Total RNA was extracted from cells using the EZNA HP Total RNA Kit (Omega). RNA was reverse transcribed using the iScript cDNA Synthesis Kit (Bio-Rad). Quantitative PCR was performed using SYBR Green qPCR Master Mix (Fisher) on the BioRad CFX96 Touch Real-Time PCR Detection System (BioRad) with the following cycling parameters: 95 °C for 5 min, 95 °C for 10 s, 60 °C for 20 s, 72 °C for 30 s, repeat from step two 39x. The Cq values obtained for *CARM1* for each sample were normalized to their own *GAPDH* levels.

*CARM1* Forward: TCGCCCTCTACAGCCATGA
*CARM1* Reverse: CACACGGCTGCACTCTGTCT
*GAPDH* Forward: AGACATCTAAGGTTCCAGTATGAC
*GAPDH* Reverse: ATCGTCCCATTTGATGTTAGAG

### Colony Formation Assay

MCF7 cells were seeded 300 cells/well in 400 μL of DMEM into the wells of a 48-well plate. MDA-MB-231 cells were seeded 200 cells/well in 400 μL of RPMI into the wells of a 48-well plate. Cells were treated with DMSO, 3e, kusunokinin, or exostemin for 2 weeks. Media and compounds were refreshed after 1 week. Formed colonies were fixed in 100% methanol for 20 min in −20 °C. Fixed colonies were stained with 0.1% crystal violet in 10% ethanol for 20–30 min. Stained colonies were imaged, then dye was resolved in 10% acetic acid for 20–30 min. Absorbance was read at 570 nm on the Victor x5 2030 Multilabel Reader (PerkinElmer).

### Migration Assay

MDA-MB-231 cells were grown in 6 cm plates and treated with DMSO, kusunokinin, or exostemin for 24 h. Treated cells (7.5 × 10^4^) in 200 μL serum-free RPMI were seeded into Transwell inserts with 8.0 μM pore size. Drug treatment was added to the wells of a 24-well plate were in 800 μL RPMI with 10% FBS. Transwells were inserted into the 24-well plate with RPMI, then incubated at 37 °C for 16 h. After incubation, cells on the upper surface of the Transwell were removed with cotton swabs. Migrated cells were fixed in 3.7% formaldehyde for 2 min at room temperature, then fixed in 20% methanol at −20 °C for 20 min. The fixed cells were stained with 1% crystal violet in 20% methanol for 20–30 min. Membranes were imaged, then resolved in 10% acetic acid for 20 min. Absorbance was read at 570 nm on the Victor x5 2030 Multilabel Reader (PerkinElmer).

### Collection, Extraction, and Isolation

#### *Myristica fatua*: Collection, Extraction, and Isolation

The leaves of *M. fatua* were collected Papua New Guinea in October 1988 under the contract through the New York Botanical Gardens for the National Cancer Institute. The plant was taxonomically identified by W. Meijer, and a voucher specimen (Q66O6631) was deposited at the Smithsonian Institution, Washington, DC. The dried ground leaves (525.0 g) were extracted with MeOH/DCM (1:1) to yield 38.8 g of the crude organic extract (N13117). Further fractionation of 2.5 g of this crude extract on a Teledyne ISCO CombiFlash Rf 200 using C_8_ (20 g) column eluting sequentially with H_2_O/MeOH (95:5) to yield 118.7 mg of fraction 1, H_2_O/MeOH (80:20) to yield 30.5 mg of fraction 2, H_2_O/MeOH (60:40) to yield 50.8 mg of fraction 3, H_2_O/MeOH (40:60) to yield 59.6 mg of fraction 4, H_2_O/MeOH (20:80) to yield 467.2 mg of fraction 5, MeOH to yield 253.8 mg of fraction 6, and finally with MeCN to yield 972.7 mg to yield fraction 7. Based on the LCMS analysis, fraction 5 (467.2 mg) enriched in compound **1** was subjected to preparative HPLC using a Phenomenex Kinetex C_18_ (5 μm, 100 Å, 150 × 21.2 mm) column at a flow rate of 10 mL/min. A linear gradient from 40% MeCN [0.1% FA] to 60% H_2_O/MeOH [0.1% FA] over 34 min, followed by a steep gradient to MeCN [0.1% FA] over 1 min, and finally an isocratic hold at MeCN [0.1% FA] for 5 min, was used for the separation. Fractions were collected at 30 s increments. Fractions 30–36 (103.4 mg) were further purified on a semipreparative HPLC utilizing a Phenomenex kinetex C_18_ (5 μm, 100 Å, 250 × 10 mm) at a flow rate of 3.8 mL/min. A linear gradient elution from 40% MeCN [0.1% FA] to 60% H_2_O/MeOH [0.1% FA] over 20 min, followed by a rapid 1 min gradient to MeCN [0.1% FA] and hold at isocratic MeCN conditions for additional 5 min, yielded >95% pure compound **1** (28.0 mg,1.1% yield).

#### General Experimental Procedures

NMR spectra were recorded at 25 °C on a Bruker Avance III HD spectrometer, equipped with a 5 mm TCI Prodigy Cryo-Probe operating at a frequency of 600 MHz. The spectra were calibrated to residual solvent signals at δ_H_ 3.30 for methanol-*d*_4_ and δ_H_ 7.25 for chloroform-*d*_1_. NMR FID processing and data interpretation was done using MestReNova software, version 15.0. Analytical liquid chromatography spectra were recorded on an Agilent 1260 Infinity II UHPLC system coupled to an Agilent 6545 QToF equipped with a dual AJS ESI source, and a Sedex 100 LT ELSD detector. A Kinetex C_18_ column (50.0 × 2.1 mm, 1.7 μm, 100 Å Phenomenex) was used. The mobile phase consisted of H_2_O + 0.1% FA (A) and MeCN + 0.1% FA (B). The gradient used was maintained at 95:5 (A:B) for 0.5 min, from 95:5 to 0:100 (A:B) for 8.0 min, maintained at 0:100 (A:B) for 0.5 min, from 0:100 to 95:5 (A:B) for 0.5 min and then equilibrated in a postrun at 95:5 (A:B) during 1.0 min. All compounds tested were at greater than 95% purity as determined by ^1^H NMR and LCMS at UV wavelengths of 210 and 254 and an evaporative light scattering detector (ELSD).

#### Kusunokinin (1)

Clear oil; NMR spectroscopic data in agreement to those previously reported;^32^ HRESIMS *m*/*z* [M + H]^+^ 371.1504 (calcd for C_21_H_23_O_6_^+^ 371.1489).

#### *Exostema caribaeum*: Collection, Extraction, and Isolation

The plant *E. caribaeum* was collected Puerto Rico in 1988 under the contract through New York Botanical Gardens for the National Cancer Institute. The plant was taxonomically identified by N. Trushell, and a voucher specimen (Q65 V209) was deposited at the Smithsonian Institution, Washington, DC. The dried and ground wood of stems (280 g) was extracted with MeOH/DCM (1:1) to yield 31.5 g of the crude organic extract (N13325). A portion of the organic extract (600 mg) was prefractionated on C_8_ SPE (8 g) eluting sequentially with H_2_O/MeOH (95:5) to yield 33.3 mg of fraction 1, H_2_O/MeOH (80:20) to yield 21.4 mg of fraction 2, H_2_O/MeOH (60:40) to yield 36.5 mg of fraction 3, H_2_O/MeOH (40:60) to yield 208 mg of fraction 4, H_2_O/MeOH (20:80) to yield 140.8 mg of fraction 5, MeOH to yield 36.5 mg of fraction 6, and MeOH/MeCN (50:50) to yield 17.3 mg of fraction 7. A portion of combined fractions 5, 6, and 7 (235 mg) were further separated on reversed phase HPLC. HPLC separations were performed on a Phenomenex Onyx Monolithic C_18_ [100 × 10 mm] column at a flow rate of 3.8 mL/min with the following conditions: an initial isocratic hold at 70% H_2_O (0.1% FA)/30% MeCN (0.1% FA) from 0 to 1.5 min, followed by a linear gradient to MeCN (0.1% FA) over 7.5 min, an isocratic hold at MeCN (0.1% FA) for 3.5 min, For each HPLC run, 5 mg of material was injected, and 22 fractions were collected in 30 s increments between 1.3 and 12.3 min. Combined fractions 9, 10, and 11 were further purified on a Kinetex C8 [5 μm, 100 Å, 150 × 21.2 mm] column at a flow rate of 10 mL/min with the following conditions: an initial isocratic hold at 95% H_2_O (0.1% FA)/5% MeCN (0.1% FA) from 0 to 5 min, followed by a linear gradient to 50% H_2_O (0.1% FA)/50% MeCN (0.1% FA) over 45 min, and an isocratic hold at 50% H_2_O (0.1% FA)/50% MeCN (0.1% FA). Fractions were collected in 30s increments, starting at *t* = 5 min. Fraction 86 yielded exostemin (2.2 mg, 0.6% yield).

#### Exostemin (3)

Clear oil; NMR spectroscopic data in agreement to those previously reported;^22^ HRESIMS *m*/*z* [M + H]^+^ 329.1023 (calcd for C_18_H_17_O_6_^+^ 329.1020).

## Supplementary Material



## Data Availability

The HRMS and NMR data for the isolated natural products has been deposited in the Harvard Dataverse (dataverse.harvard.edu) and can be found at 10.7910/DVN/DIHYJO.

## Notes

Luciferase and cell viability assays of lead fractions, IC50 determinations of bafilomycin A and TAK-243, cross treatment of lysosomal inhibitor with exostemin A and ubiquitin-activating enzyme inhibitor with kusunokinin, HPLC traces of exostemin and kusunokinin (PDF)

## Abbreviations

AKR1B: aldo-keto reductase family 1 member B1
CARM1: coactivator-associated arginine methyltransferase 1
CSF1R: colony stimulating factor 1 receptor
LYTAC: lysosome-targeting chimera
NPNPD: National Cancer Institute Program for Natural Product Discovery
PRMT: protein arginine methyltransferase
PROTAC: proteolysis-targeting chimera
TPD: targeted protein degraders
UPS: ubiquitin-proteasome system
