# Supplementary material for: Utilizing the HiBiT System to Identify CARM1 Degraders for Targeted Cancer Therapy
Source: J Med Chem. 2025 Dec 26;69(1):134–45. doi: 10.1021/acs.jmedchem.5c01863 (PMC12794155; doi:10.1021/acs.jmedchem.5c01863)
Supplement: Supplementary file 1 [file jm5c01863_si_001.pdf]

Supporting Information

Utilizing the HiBiT system to identify CARM1 degraders for targeted cancer therapy

Megan Bacabac[1‡], Mingshan Hu[1‡], Fabao Liu[1], Eui-Jun Kim[1], Tanja Grkovic[2,3], Rohitesh Kumar[4], Rhone K. Akee[4], Isaac Hayes[1], Ramesh Mudududdla[5,6], Yidan Wang[1], Mason McGuire[1], Weiping Tang[5,6], Barry R O'Keefe[2,3], Tim S Bugni[5, 6], Wei Xu[1*]

1 McArdle Laboratory for Cancer Research, University of Wisconsin-Madison 53705

2 Natural Products Branch, Developmental Therapeutics Program, Division of Cancer Treatment and Diagnosis , National Cancer Institute , Frederick , Maryland 21702-1201, United States.

3 Molecular Targets Program, Center for Cancer Research , National Cancer Institute , Frederick , Maryland 21702-1201, United States.

4 Natural Products Support Group, Leidos Biomedical Research, Inc., Frederick National Laboratory for Cancer Research Frederick, Maryland 21702-1201, United States.

5 Lachman Institute for Pharmaceutical Development, School of Pharmacy, University of Wisconsin-Madison 53705

6 Pharmaceutical Sciences Division, University of Wisconsin-Madison 53705

‡ These authors contributed equally.

Corresponding Author

Wei Xu: wxu@oncology.wisc.edu

Table of Contents

Figure S1. Lead fractions decrease HiBiT signal but do not significantly decrease cell viability. S3

Figure S2. IC50 determination of bafilomycin A. S4

Figure S3. IC50 determination of TAK-243. .... S5

Figure S4. Cross treatment of lysosomal inhibitor with exostemin and ubiquitin-activating enzyme inhibitor with kusunokinin. S6

HPLC traces of compounds exostemin and kusunokinin (Figure S5-8). S7

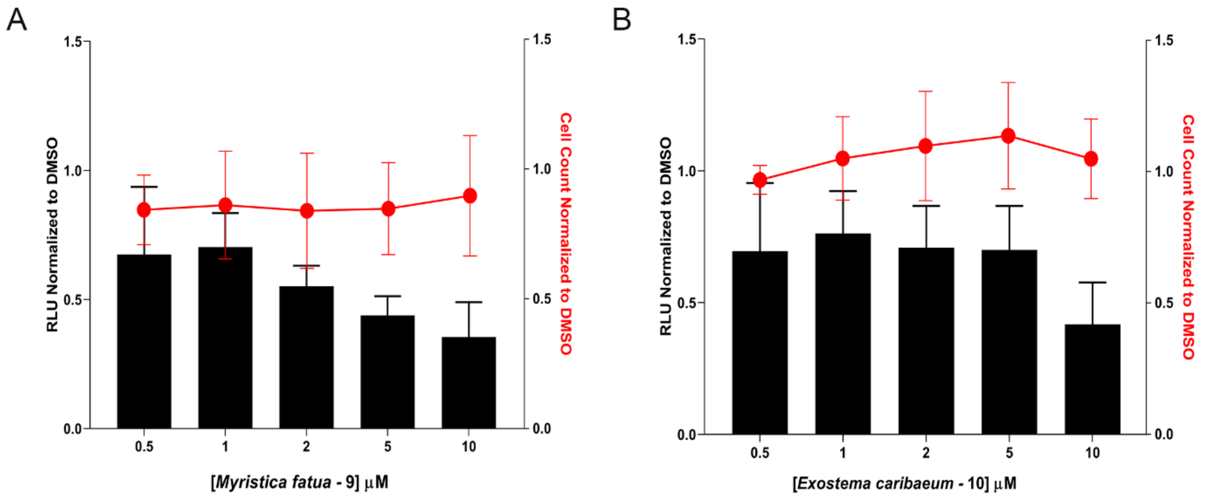

Figure S1. Lead fractions decrease HiBiT signal but do not significantly decrease cell viability. MCF7-HiBiT-CARM1 cells were treated with indicated doses *of Myristica fatua* - 9 (A) *or Exostema caribaeum* - 10 (B) for 48h. HiBiT-CARM1 levels were assessed by a luciferase assay (black bars) and cell viability was assessed by cell counting (red dots).

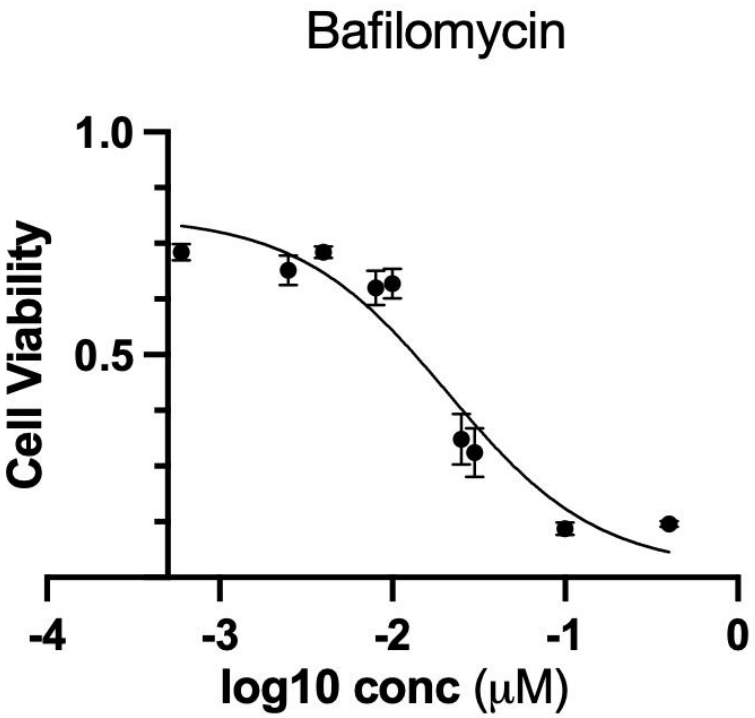

Figure S2. IC50 determination of bafilomycin A. Cell viability curves of MCF7-HiBiT-CARM1 cell lines treated with Bafilomycin at various concentrations for 72 hr. IC_{50}=0.02μM.

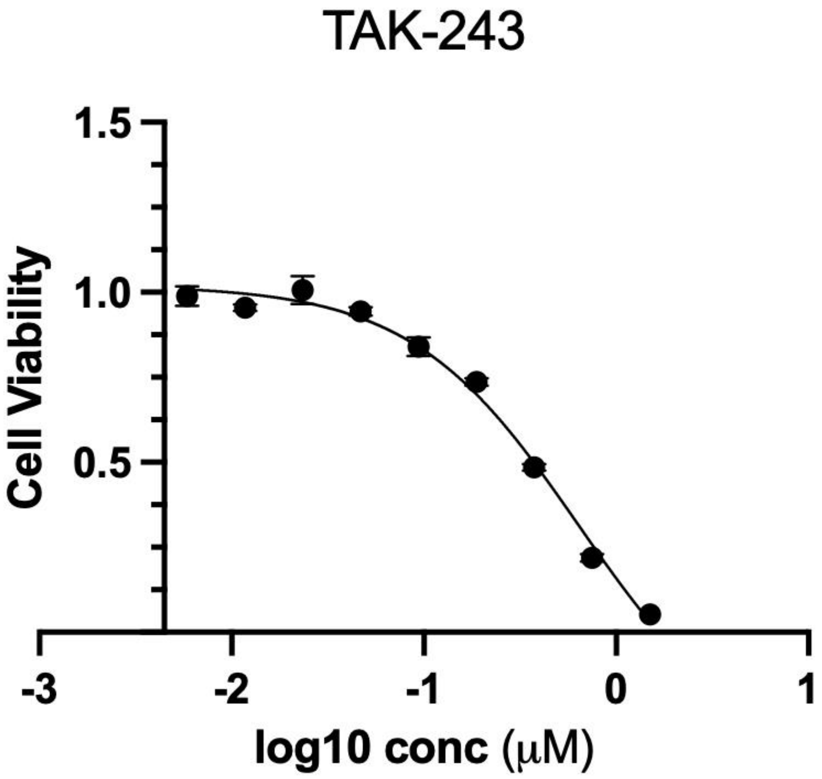

Figure S3. IC50 determination of TAK-243. Cell viability curves of MCF7-HiBiT-CARM1 cell lines treated with TAK-243 at various concentrations for 72 hr. IC_{50}=0.6μM.

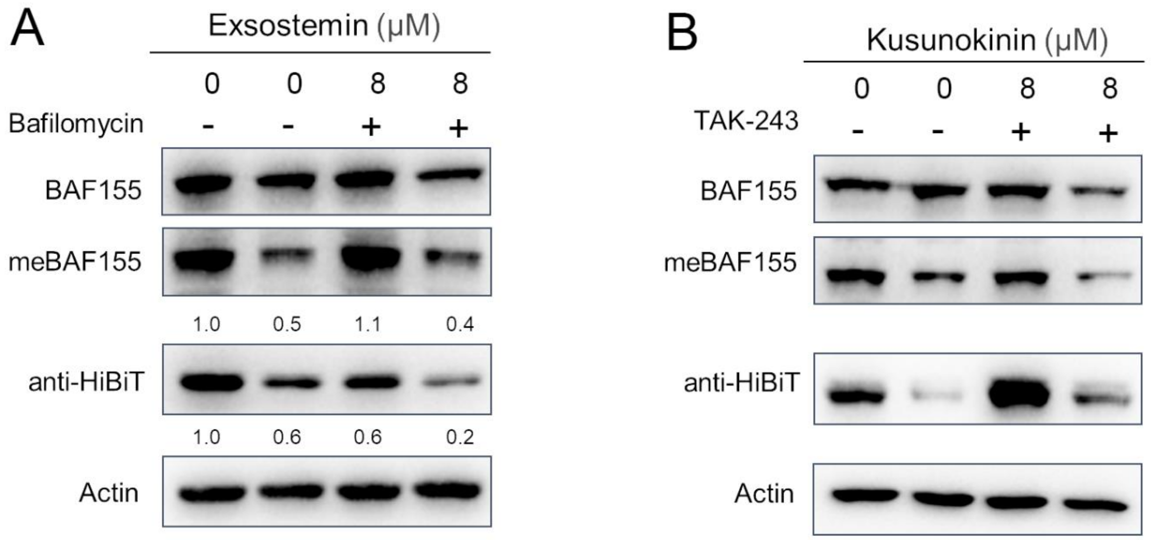

Figure S4. Cross treatment of lysosomal inhibitor with exostemin and ubiquitin-activating enzyme inhibitor with kusunokinin. A) MCF7-HiBiT-CARM1 cells were treated for 72 hours with 8μM exostemin with or without 0.02μM bafilomycin. BAF155, meBAF155, and HiBiT-CARM1 levels were assessed by immunoblotting. B) MCF7-HiBiT-CARM1 cells were treated for 72 hours with 8μM kusunokinin with or without 0.03μM TAK-243. BAF155, meBAF155, and HiBiT-CARM1 levels were assessed by immunoblotting.

HPLC traces of compounds exostemin and kusunokinin.

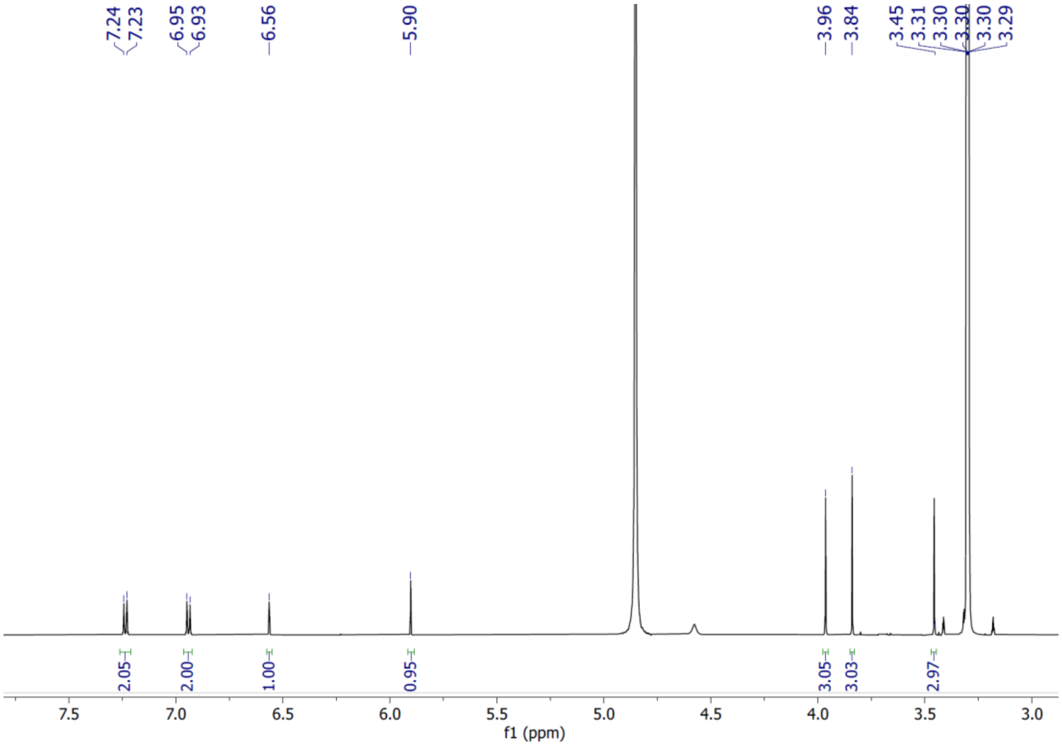

Figure S5. ^{1}H NMR spectrum of exostemin in methanol-d_4 at 600 MHz.

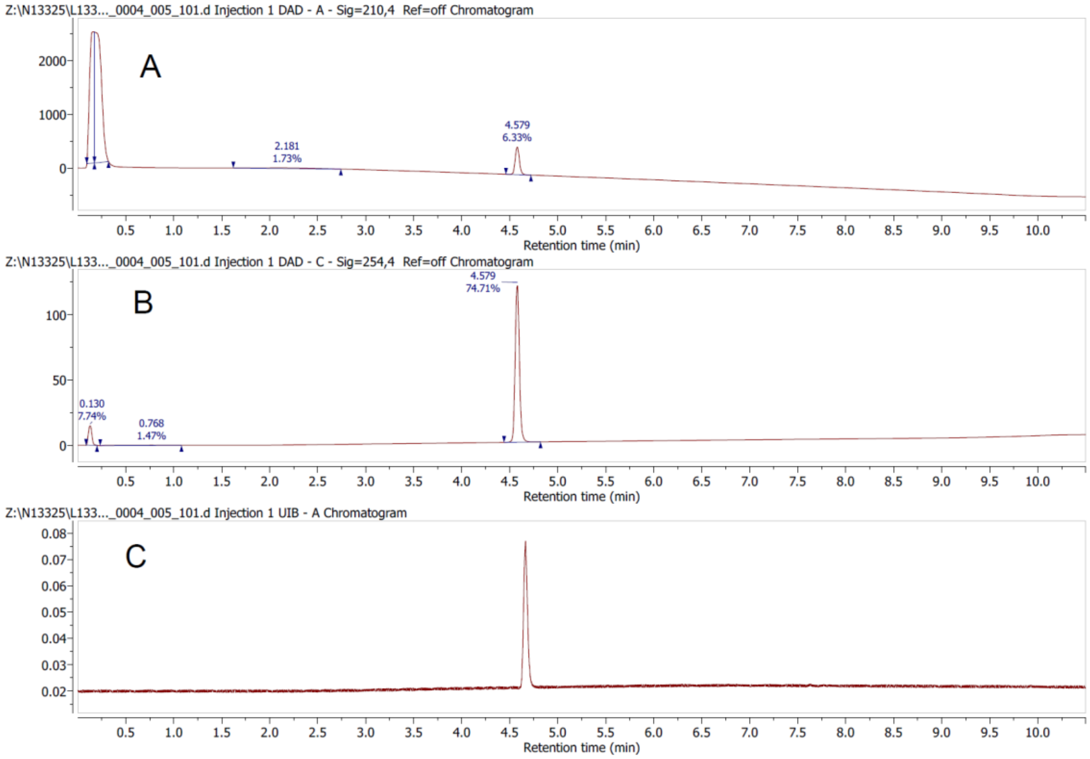

Figure S6. LCMS spectrum of exostemin. A) LC trace at 210 nm; B) LC trace at 254 nm; E) evaporative light scattering detector trace.

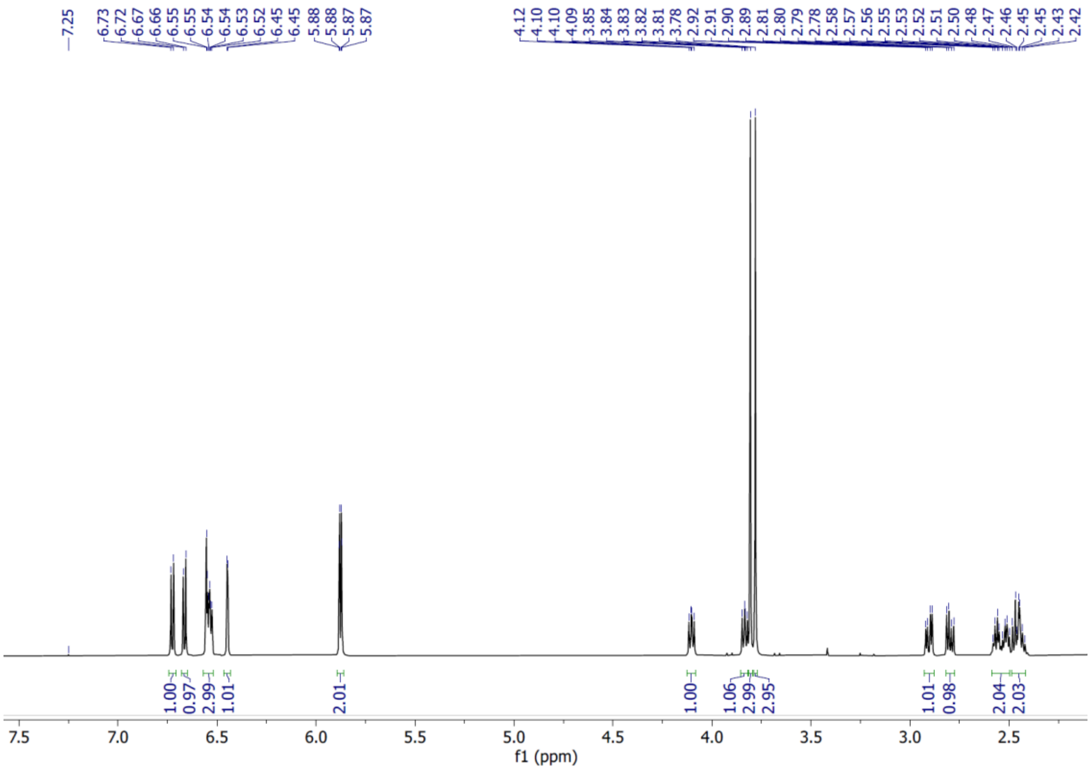

Figure S7. ^{1}H NMR spectrum of kusunokinin in chloroform-d_1 at 600 MHz.

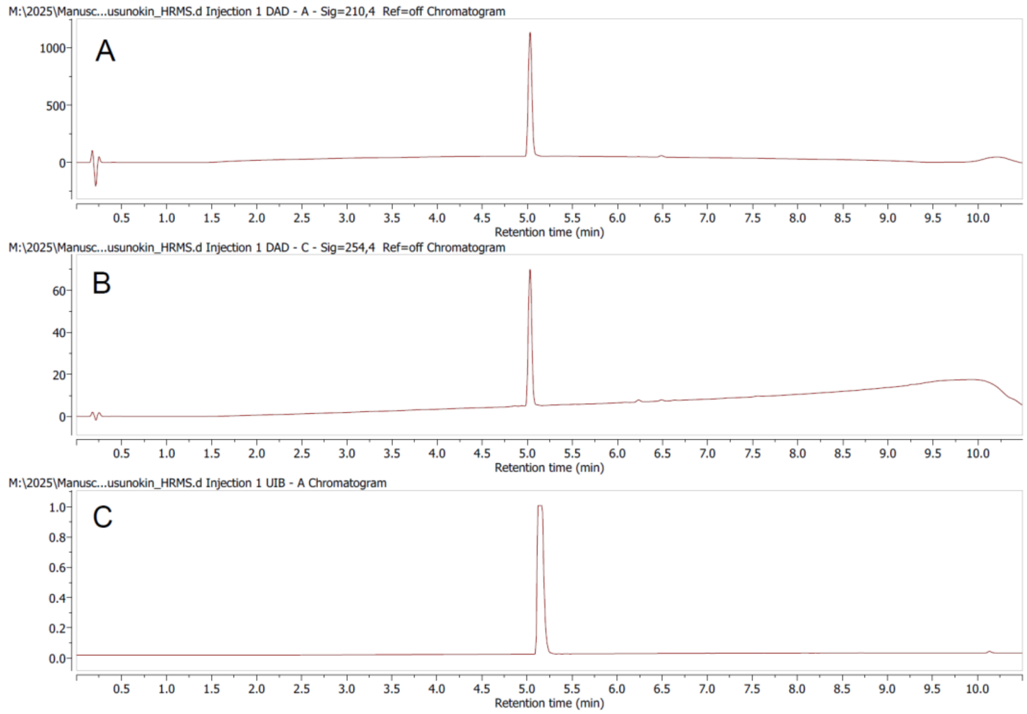

Figure S8. LCMS spectrum of kusunokinin. A) LC trace at 210 nm; B) LC trace at 254 nm; E) evaporative light scattering detector trace.
